# Snapshot Surveys for Lake Monitoring, More Than a Shot in the Dark

**DOI:** 10.3389/fevo.2018.00201

**Published:** 2018

**Authors:** Evanthia Mantzouki, Meryem Beklioǧlu, Justin D. Brookes, Lisette Nicole de Senerpont Domis, Hilary A. Dugan, Jonathan P. Doubek, Hans-Peter Grossart, Jens C. Nejstgaard, Amina I. Pollard, Robert Ptacnik, Kevin C. Rose, Steven Sadro, Laura Seelen, Nicholas K. Skaff, Katrin Teubner, Gesa A. Weyhenmeyer, Bastiaan W. Ibelings

**Affiliations:** 1 Department F.-A. Forel for Environmental and Aquatic Sciences, University of Geneva, Geneva, Switzerland,; 2 Limnology Laboratory, Department of Biological Sciences, Middle East Technical University, Ankara, Turkey,; 3 Department of Environmental Biology, The University of Adelaide, Adelaide, SA, Australia,; 4 Department of Aquatic Ecology, Netherlands Institute of Ecology (NIOO-KNAW), Wageningen, Netherlands,; 5 Department of Environmental Sciences, Wageningen University & Research, Wageningen, Netherlands,; 6 Center for Limnology, University of Wisconsin-Madison, Madison, WI, United States,; 7 Rubenstein Ecosystem Science Laboratory, University of Vermont, Burlington, VT, United States,; 8 Department of Experimental Limnology, Leibniz Institute of Freshwater Ecology and Inland Fisheries, Stechlin, Germany,; 9 Faculty of Mathematics and Natural Sciences, Institute of Biochemistry and Biology, Potsdam University, Potsdam, Germany,; 10 Office of Water, US Environmental Protection Agency, Washington, DC, United States,; 11 WasserCluster Lunz, Biologische Station GmbH, Lunz am See, Austria,; 12 Department of Biological Sciences, Rensselaer Polytechnic Institute, Troy, NY, United States,; 13 Department of Environmental Science and Policy, University of California, Davis, Davis, CA, United States,; 14 Department of Fisheries and Wildlife, Michigan State University, East Lansing, MI, United States,; 15 Department of Limnology and Bio-Oceanography, Faculty of Life Sciences, University of Vienna, Vienna, Austria,; 16 Department of Ecology and Genetics/Limnology, Uppsala University, Uppsala, Sweden

**Keywords:** multi-lake snapshot surveys, lake monitoring, Nyquist-shannon sampling theorem, space-for-time substitution, phytoplankton ecology

## INTRODUCTION–WHY DO WE MONITOR?

Environmental degradation and loss of ecosystem services due to anthropogenic activities are an issue of global concern (Cardinale et al., 2012). Lakes act as effective sentinels of environmental change as they respond to atmospheric, terrestrial, and hydrological processes (Williamson et al., 2008). Understanding lake dynamics can help determine the scale and frequency of occurring changes, establish control measures and maintain ecosystem integrity. Thus, monitoring is necessary, but it is rendered impossible since there are over 117 million lakes globally (Verpoorter et al., 2014).

Monitoring strategies that range from long-term time-series on individual lakes to short-term snapshot surveys of up to thousands of lakes from disparate locations serve different purposes and cover different temporal- and spatial-scales of ecological phenomena. For example, phytoplankton dynamics can be driven by long-term environmental change (Monchamp et al., 2016), inter-annual variability (Anneville et al., 2004), seasonal succession (Sommer et al., 2012), and diel changes (Ibelings et al., 1991). To efficiently capture their temporal and spatial variability, the appropriate monitoring strategy needs to be chosen (Supplementary Table 1).

Implementation of long-term monitoring strategies face many challenges. Water quality monitoring programs are usually restricted to priority ecosystems (e.g., socio-economically important or “easier to reach”), creating geographical biases in observations which may not be representative of broader regions or even nearby waterbodies (Ruiz-Jaen and Aide, 2005). Thus, long term monitoring alone is insufficient. To develop a global understanding of environmental response, we need to consider both the sampling frequency and efficiency of monitoring. Combining different monitoring strategies such as automated high frequency and multi-lake snapshot surveys, may allow studying numerous lakes over many years and at the continental or even global scale.

Here, we explore the advantages and disadvantages of widely used sampling strategies. We focus on multi-lake snapshot surveys and discuss the limitations of the approach. This strategy allows broad spatial coverage, while remaining affordable. We use mostly phytoplankton examples, because of its rapid response to environmental change (Carpenter et al., 2006).

## DIFFERENT MONITORING STRATEGIES

### Long-Term Monitoring From Routine (Discrete) Sampling

Long-term monitoring from routine (discrete) sampling—typically bi-weekly to monthly—addresses ecosystem change under environmental pressure over time by measuring both coarse and fine-resolution responses (e.g., phytoplankton taxonomy) and environmental drivers (e.g., nutrients) that cannot be sampled with automated or remote sensing approaches. The resulting datasets can elucidate long-term impacts on lakes such as eutrophication (North et al., 2014). Such datasets contributed to developing and validating ecological theories, e.g., the alternative stable state theory (Scheffer and van Nes, 2007), which was successfully implemented in lake restoration programs (Ibelings et al., 2007). Long-term sampling may, however, introduce data inconsistencies over time, due to changes in the sampling protocols, analysis methods and staff employed (Straile et al., 2013). Also, the frequency of routine sampling associated with long-term monitoring does not necessarily assure correct capture of lake processes.

### Long-Term Monitoring From Automated High-Frequency Sampling

Long-term monitoring from automated high-frequency sampling allows characterization of fine-scale temporal dynamics. High-frequency sampling can reveal the buildup and break-down of episodic phytoplankton blooms that cannot be captured with routine sampling (Pomati et al., 2011). Grassroots initiatives like GLEON, support the use of automated high-frequency lake stations worldwide (Weathers et al., 2013). In most cases the characterization of phytoplankton dynamics remains limited to chlorophyll-a measurements from fluorescence sensors. Methods like flowcytometry (Pomati et al., 2011) or image analysis (Sosik and Olson, 2007) are expensive, while data handling requires qualified personnel. Affordable fluorescence probes (e.g., Fluoroprobe-Moldaenke, Germany) that measure pigments of different phytoplankton classes could be an alternative but offer limited taxonomic information to determine community dynamics.

### Remote Sensing

Remote sensing provides broad spatial coverage and relatively frequent images. The Landsat satellites have operated since 1972, with a 16-day location-specific revisiting time and spatial resolution of 30–79 m. The newly launched Sentinel satellites have a 5-day revisiting time and spatial resolution of 10–60 m (Toming et al., 2016). The advanced radiometric resolution of Sentinel satellites along with published band ratio algorithms that estimate chlorophyll-a, colored dissolved organic matter and dissolved organic carbon, make them highly suitable for monitoring lakes (Toming et al., 2016). Remote sensing can, however, be limited by cloud cover (Ibelings et al., 2003), and thus needs to be integrated in a multiplatform monitoring approach (Vos et al., 2003) with airborne based remote sensing and good quality *in-situ* data for ground truthing.

### Disparate Data

The assembly of multi-lake datasets from disparate sources is flourishing. Disparate data provide a broader representation of environmental change at larger spatial-scales and complementary temporal coverages. International collaborations support such efforts and promote open science to achieve deeper understanding of lake ecosystems globally (Soranno and Schimel, 2014). LAGOS-NE comprises thousands of lakes with diverse geographic conditions and land use histories (Soranno et al., 2017). Disparate data have resulted in important insights into lake functioning (e.g., O’Reilly et al., 2015). Integrating disparate data, however, is a great challenge. Lack of standardization in data protocols and heterogeneity in data formats and units necessitates manual integration (Soranno et al., 2017). Such data inconsistencies should be resolved to successfully attribute environmental change to regional characteristics and not to protocol differences (Moe et al., 2008). Trustworthy databases of disparate data require time and qualified specialists, making it a laborious and costly project (Soranno et al., 2017).

### Multi-Lake Snapshot Surveys (MLSS)

Multi-lake Snapshot Surveys (MLSS) sample many lakes across large geographic distances, only once, within a predefined period. We define snapshot sampling as the acquisition of biological, chemical, and physical parameters at intervals that violate the Nyquist-Shannon sampling theorem. According to this theorem, in order to fully capture a phenomenon, we need to sample at a Nyquist rate which exceeds twice the maximum component frequency (i.e., Nyquist frequency) of the sampled function (Marcé et al., 2016). If for example we study diel re-positioning of algal communities in the water column—which is the outcome of processes that operate on short time-scales—we should sample at hourly intervals (Ibelings et al., 1991). Inadequate sampling rate may result in a loss but also a distortion of sampled information (i.e., aliasing—Jerri, 1977).

## ADVANTAGES OF THE MLSS

### Status Assessment of Freshwater Systems Across Large Geographical Areas

MLSS mostly use standard protocols that minimize sampling effort per lake without sacrificing data quality (Mantzouki and Ibelings, 2018; Pollard et al., 2018). Hence, numerous lakes can be sampled across large geographical areas to frequently assess ecological status (e.g., EU Water Framework Directive, Nordic freshwater inventory—Skjelkvale et al., 2001) and provide ecological understanding. For example, the South American Lake Gradient Analysis (SALGA) investigated the role of temperature on cyanobacterial occurrence in shallow lakes along a latitudinal gradient (Kosten et al., 2012). The National Lake Assessment (NLA) of the US Environmental Protection Agency (US-EPA), sampled over 1,000 lakes in 2007 and 2012 (Pollard et al., 2018) to study water quality (Rigosi et al., 2014), food web issues (Doubek and Carey, 2017) and changes over time (Leech et al., 2018). The European Multi-Lake Survey (EMLS) sampled 400 lakes to investigate how temperature and nutrients determine variation in algal and cyanobacterial biomass and toxins (Mantzouki et al., 2018).

### Standardized Data Across Large Geographical Areas

MLSS can produce highly comparable datasets, with uniform, synchronic data. Data curators can more easily manipulate the collected data (e.g., outliers’ identification) and perform better quality assurance and control. Thus, data integration can be performed with high fidelity. For complete data integration, data collectors should strictly follow standardized procedures. In the EMLS, representatives from 27 European countries jointly defined the research questions and developed the protocols, during a 3-day training school. The trainees obtained hands-on experience in the agreed protocols and then disseminated the information at the national level. Centralization of key analyses (done by one person on one machine) was also a significant step to assure successful data integration (Mantzouki and Ibelings, 2018).

Selection of MLSS lakes is based on sound scientific criteria. The NLA uses a Generalized Random Tessellation Stratified Survey Design (GRTS) which is a spatially-balanced probabilistic design that avoids clumping of sampling locations (Kincaid et al., 2013). MLSS typically engage numerous data collectors that sample many lakes simultaneously. Confounding effects of seasonality can thus be avoided. For example, the EMLS sampled during the locally warmest 2-week period to focus on cyanobacterial blooms—a distinct feature of summer phytoplankton (Sommer et al., 2012).

### Cost and Time Efficiency

Cost and time efficiency is an important advantage of MLSS that can enable global participation and thus investigate landscape-related variation in lakes at large spatial-scale (Sadro et al., 2012). The one-time sampling in a MLSS reduces costs and permits the sampling of numerous lakes. MLSS are particularly suited to grassroots approaches that typically have limited financial means and rely on the motivation and dedication of many scientists from different countries. This low-cost approach allows the participation of researchers and institutes with different levels of funding and equipment, since it does not rely on expensive instrumentation. Because the individual sampling effort in MLSS is not particularly time demanding, numerous environmental parameters can be sampled and analyzed at a higher analytical resolution. Thus, MLSS can provide a deeper insight into specific ecological relationships (NLA- and EMLS-related references) which cannot be achieved by high-frequency monitoring strategies.

### Space-for-Time Substitution (SfTS)

Frequently, MLSS aim to capture environmental differences at geographical gradients to provide insight into impacts of future environmental change. MLSS may use space-for-time substitution (SfTS) (Blois et al., 2013) to study present-day spatial phenomena instead of long-term records that often are unavailable (Pickett, 1989). Sampling numerous lakes is needed for an adequate SfTS. The statistical power generated by sampling many different lakes can overcome the risk of gaining idiosyncratic results from long-term monitoring of only a few lakes.

To develop reliable SfTS we need to consider that drivers of temporal change are not necessarily constant across various time-scales. Drivers of large-scale spatial variation rather than of shorter-term temporal variation may be better predictors of long-term climatic change in ecosystems. For example, in grassland communities, geographic rather than temporal variation in annual precipitation and plant community structure better predicted climate-driven changes in precipitation (Adler and Levine, 2007). See also Taranu et al. (2012) on the importance of scale on temporal change.

Temporal drivers of lake change may also differ from spatial drivers, at a short temporal-scale (<20 years) probably because the time-scale (rate and persistence) of change differs in space and time (Weyhenmeyer, 2009). Spatial data may capture the lake’s history over time, i.e., the long-term impact of an environmental predictor but not its short-term impact. For instance, dissolved organic carbon (DOC) and partial pressure of CO_2_ (pCO_2_) are related at the spatial-scale (Lapierre and Giorgio, 2012) but fast processes such as flushing-rate can result in a decoupling of the two parameters on a temporal-scale (Nydahl et al., 2017). However, long-term and spatial-scale ice breakup data showed similar patterns of temperature effects on ice-off timing (Weyhenmeyer et al., 2004). Similarly, in 1,041 boreal lakes the correlation of chemical variability with increased temperature was consistent across space and time (Weyhenmeyer, 2009). Climate change is emerging as a major driver of both spatial and temporal variation in lake dynamics (Weyhenmeyer, 2009), thus a SfTS may be a suitable solution to predict change.

## CONCLUSIONS

There are obvious trade-offs between monitoring strategies and no single strategy can provide answers to all research questions, lake management, or water governance requirements. An ideal approach might be to organize a yearly MLSS, with both previous and new lakes sampled every year and revisited at a certain time-interval to assess changes in the lake status at a broad spatial-scale. Additionally, time-series from key lakes could be obtained to develop tailor-made SfTS predictive models. We argue that MLSS, if properly designed and executed, comprise a promising solution for assessing lakes globally, ensuring data integration and engaging researchers, managers, policy makers, and citizens (Weyhenmeyer et al., 2017). For a successful MLSS, sampled environmental parameters should be carefully chosen to ensure a reliable SfTS. Numerous lakes, well-spread geographically, should be sampled to cover wide environmental gradients. If the right pre-conditions are met and a standardized sampling plan is established, then MLSS can be an accurate and cost-efficient solution. International, grassroots efforts are increasingly establishing automated high-frequency monitoring stations worldwide. These efforts, along with more MLSS initiatives, could eventually contribute toward a better understanding of both spatial and temporal environmental patterns in lakes.

## Supplementary Material

Sup 1

## Abbreviations:

MLSS: Multi-Lake Snapshot Surveys
SfTS: Space for time substitution
