# Supplementary material for: Snapshot Surveys for Lake Monitoring, More Than a Shot in the Dark
Source: Front Ecol Evol. Author manuscript; Available in PMC 2020 Mar 17. (PMC7077876; doi:10.3389/fevo.2018.00201)
Supplement: Sup 1 [file NIHMS1052921-supplement-Sup_1.pdf]

Table 1. Characteristics of the five main lake monitoring strategies (Routine sampling, automated high-frequency sampling, remote sensing, disparate data and snapshot sampling) addressing the scale that they can cover (temporal vs. spatial); the investment in time, money, personnel and equipment; the potential outcome with regards data integration, accuracy, efficiency collaboration and data sharing; and potential caveats.

	Rank	Routine Sampling	Automated High Frequency	Remote Sensing	Disparate Data	Snapshot Sampling
Scale	Temporal scale	✔ (orange)	✔ (green)	✔ (orange)	✔ (orange)	✔ (orange)
	Spatial scale	✔ (orange)	✔ (orange)	✔ (green)	✔ (green)	✔ (green)
Investment	Time	Long-term effort	Long-term effort	Long-term effort	Long-term effort	Short-term effort
	Money	Relatively expensive	Cheap to expensive	Relatively cheap	Expensive	Relatively cheap
	Personnel	Team effort	Individual-team effort	Team effort	Team effort	Individual-team effort
	Equipment	Low - High tech Long-term usage	Low - High tech Long-term usage	High tech Long-term usage	High tech Long-term usage	Low tech Short-term usage
Outcome	Data Integration	★★★☆☆	★★★☆☆	★★★★☆	★★☆☆☆	★★★★☆
	Accuracy	★★☆☆☆	★★★★☆	★★★★☆	★★★☆☆	★★★★☆
	Efficiency	★★☆☆☆	★★★★☆	★★★☆☆	★★★☆☆	★★★★★
	International Collaboration	Not necessary	Not necessary	Necessary	Necessary	Mostly necessary
	Data Sharing	Rarely open access	Increasingly open access	Mostly open access	Mostly open access	Mostly open access
	Caveats	Human error, seasonality	Lack of funding, instrument failure	Weather conditions	Incomplete data integration	Large spatial coverage required

Legend

Temporal and Spatial scale – green check marks indicate strategies that we deem to be particularly strong in this respect while orange check marks indicate strategies with a potential to cover temporal or spatial scales.
Time – the amount of time required to obtain a comprehensive dataset, based on the research question. Snapshot sampling is attractive based upon this criterion, yielding information at shorter time-scales than most other methods.
Money – the funds that the end-users need to invest to build or have access to the dataset. Remote sensing for instance is expensive to get up and running, but for end-users in academia the images are often available at no to low cost.
Personnel – the amount of (trained) employees needed to acquire data and maintain meaningful datasets. Is it typically a team effort or could individuals or small groups manage by themselves?
Equipment – the type of equipment needed to acquire data in a consistent manner, being technologically advanced or not, and remaining functional for longer or shorter periods.
Data Integration – how easily can datasets from different sampling efforts be combined into an integrated dataset?
Accuracy – It is hard to award distinctions for this criterion, different methods are appropriate for different types of questions
Efficiency – "Bang for the buck". The amount of scientifically valuable data obtained per unit (monetary) investment.
International Collaboration – Is international collaboration essential to create a usable dataset?
Data Sharing – Feasibility to publish datasets in an open, publicly accessible format
Caveats – potential caveats linked to e.g. research purposes not being clear, funds not being permanently available or confounding effects of seasonality for the detection of long-term trends etc.
